# COVID-19 vaccination coverage and its cognitive determinants among older adults in Shanghai, China, during the COVID-19 epidemic

**DOI:** 10.3389/fpubh.2023.1163616

**Published:** 2023-06-02

**Authors:** Lu Wei, Wu Zeng, Yangyang Huang, Guoxin Ye, Ying Chen, Ling Yang, Yuyang Cai

**Affiliations:** ^1^Department of Geriatrics, Xinhua Hospital, Shanghai Jiao Tong University School of Medicine, Shanghai, China; ^2^Department of Global Health, School of Health, Georgetown University, Washington, DC, United States; ^3^Department of Geriatrics, Shanghai Fourth People's Hospital Affiliated to Tongji University, Shanghai, China; ^4^School of Public Health, Shanghai Jiao Tong University School of Medicine, Shanghai, China

**Keywords:** COVID-19, cognition, attitude, vaccine, vaccine hesitancy, older adults

## Abstract

**Objectives:**

This study aimed to examine the coverage of coronavirus disease 2019 (COVID-19) vaccination and its cognitive determinants among older adults.

**Methods:**

A cross-sectional study was conducted using a questionnaire to conduct a survey among 725 Chinese older adults aged 60 years and above in June 2022, 2 months after the mass COVID-19 outbreak in Shanghai, China. The questionnaire covered demographic characteristics, COVID-19 vaccination status, internal risk perception, knowledge, and attitude toward the efficacy and safety of COVID-19 vaccines.

**Results:**

The vaccination rate was 78.3% among the surveyed individuals. Self-reported reasons for unwillingness to get vaccinated (multiple selections) were “concerns about acute exacerbation of chronic diseases after vaccination (57.3%)” and “concerns regarding vaccine side effects (41.4%).” Compared to the unvaccinated group, the vaccinated group tended to have a higher score in internal risk perception (*t* = 2.64, *P* < 0.05), better knowledge of COVID-19 vaccines (*t* = 5.84, *P* < 0.05), and a more positive attitude toward the efficacy and safety of COVID-19 vaccines (*t* = 7.92, *P* < 0.05). The path analysis showed that the cognitive effect on vaccination behavior is relatively large, followed by the internal risk perception, and then the attitude toward COVID-19 vaccines. The more knowledgeable the participants were about COVID-19 vaccines, the more likely they were to receive the COVID-19 vaccines. In the multivariate logistic regression, the increased coverage of COVID-19 vaccination was associated with reduced age (OR = 0.53 95% CI 0.43–0.66, *P* < 0.001), being a resident in other places than Shanghai (OR = 0.40, 95% CI 0.17–0.92, *P* < 0.05), a shorter time of lockdown (OR = 0.33, 95% CI 0.13–0.83, *P* < 0.05), a history of other vaccines (OR = 2.58, 95% CI 1.45–4.60, *P* < 0.01), a fewer number of chronic diseases (OR = 0.49, 95% CI 0.38–0.62, *P* < 0.001), better knowledge about COVID-19 vaccines (OR = 1.60, 95% CI 1.17–2.19, *P* < 0.01), and a positive attitude toward COVID-19 vaccines (OR = 9.22, 95% CI 4.69–18.09, *P* < 0.001).

**Conclusion:**

Acquiring accurate knowledge and developing a positive attitude toward COVID-19 vaccines are important factors associated with COVID-19 vaccination. Disseminating informed information on COVID-19 vaccines and ensuring efficacious communication regarding their efficacy and safety would enhance awareness about COVID-19 vaccination among older adults and consequently boost their vaccination coverage.

## Introduction

Coronavirus disease 2019 (COVID-19), caused by severe acute respiratory syndrome coronavirus 2 (SARS-CoV-2), has put a heavy toll on public health, lives, and the world economy (1, 2). As of 21 March 2023, the global tally of confirmed COVID-19 cases has surpassed 761.1 million, with the death toll exceeding 6.9 million (3). The persistent mutations of the virus serve as a stark reminder that the task of epidemic prevention remains a formidable challenge. It was reported that individuals aged 60 years and above had a five times higher risk of death after symptomatic SARS-CoV-2 infection than adults aged 30–59 years (4), suggesting that protecting older adults, a vulnerable group, is critical to saving lives during the pandemic (5).

Vaccines have been proven an extremely effective means of combating epidemics in the past (6). Since the start of the pandemic, scientists around the world have been working to develop vaccines against SARS-CoV-2 infection. COVID-19 vaccines have been tested and put into use at an unprecedented pace (7, 8). The success of various COVID-19 vaccines depends not only on the effectiveness of vaccines but also on the degree of the uptake of vaccination by the target population. An adequate uptake of COVID-19 vaccines would ensure the protection for the vaccinated population and possesses the potential to stop the pandemic through herd immunity, thereby protecting even those who are not vaccinated (9).

Vaccine willingness/hesitancy is one of the key factors determining the coverage of vaccination. Although vaccines are effective in preventing risk populations from being infected, there is still a high level of COVID-19 hesitancy, globally (10, 11). In 2015, the WHO Strategic Advisory Group of Experts on Immunization (SAGE) defined vaccine hesitancy as a “delay in acceptance or refusal of vaccination despite the availability of vaccination services” (12). Vaccine hesitancy is regarded to be responsible for the low coverage of vaccines and contributes to an increased risk of outbreaks of vaccine-preventable diseases (13). The WHO has listed vaccine hesitancy as one of the top 10 human health threats (14). The vaccine hesitancy of COVID-19 is increasing due to various reasons that are yet to be identified. Extensive research has documented some sociodemographic factors associated with COVID-19 vaccine hesitancy, including gender, race, education, political orientation, geographic location, and economic status (15, 16). Recent studies show that cognitive and psychosocial factors also affect vaccination decisions (17).

Since December 2021, the Omicron variant of SARS-CoV-2 has quickly spread throughout the world and mutated rapidly (18). Data from Hong Kong showed that 95% of COVID-19 deaths occurred in people aged 60 years, with a high fatality rate among unvaccinated older adults (19). New Zealand also experienced a similar increase in COVID-19 incidence due to the Omicron variant during the same period. However, New Zealand, with 95% of people above 60 years vaccinated with 2-dose COVID-19 vaccines, had a mortality rate that peaked at 2.1 per million population per day, which was much lower than the mortality rate of 38.0 per million in Hong Kong (20). In late February 2022, a wave of SARS-CoV-2 infection rapidly attacked Shanghai, China. Although omicron BA.2 became less virulent than the original strain, severe outcomes and high mortality had been reported among unvaccinated people in Shanghai, especially in older adults (21). These findings indicate that the risk of death from COVID-19 increases with age and reinforces the urgency to scale up the coverage of COVID-19 vaccines to protect older adults from the Omicron variant infection.

As of 7 July 2022, a total of 346,557 million doses of COVID-19 vaccines had been administered in China, with a crude coverage of 91.9% among the total population and 88.6% among people over the age of 60 years (22). However, the COVID-19 vaccine coverage among people aged 60 years and above was lower in Shanghai, with a crude coverage of 67.4% and a full coverage rate of 63.2% by 6 June 2022 (23), reflecting the existence of COVID-19 vaccine hesitancy among them. To estimate the coverage of COVID-19 vaccination and to better understand the associated factors, especially cognitive factors among older adults, we conducted a survey in June 2022, 2 months after the mass outbreak of the COVID-19 epidemic in Shanghai, China.

## Methodology

### Study design

A survey was conducted among older adults aged 60 years and above from 5 June 2022 to 20 June 2022, 2 months after the mass COVID-19 outbreak in Shanghai, China. As an economic center in China, Shanghai serves as an important transportation hub to connect to other cities and regions in the country. As of April 2022, Shanghai had adopted strict control strategies to reduce transmission and to provide early diagnosis. Schools and businesses were closed, and public transport was shut down. Most residents were not allowed to leave their community but could only go outside for an essential reason (e.g., medical emergencies). Inclusion criteria to recruit participants were (1) people aged 60 years and above living in China; (2) people without severe hearing impairment; (3) individuals with a certain cognitive ability to correctly understand the questionnaire and who could independently complete the survey; and (4) those who voluntarily agreed to participate in the present study. The study protocol was approved by the Ethics Committee of Xinhua Hospital Affiliated with Shanghai Jiao Tong University School of Medicine (Approval number XHEC-D-2022-242).

A self-designed questionnaire containing 30 items was used in this study (Appendix A). The initial draft questionnaire was developed by reviewing relevant literature (24, 25). It was then modified and finalized after consultation with experts and pre-testing the instrument. The process of the pre-testing is described later in the manuscript. The final questionnaire collected information on COVID-19 vaccination status, demographic factors, internal risk perception of the pandemic, cognition and attitude toward the COVID-19 vaccine, individuals' health status, experience with COVID-19 infections, and experience with other vaccinations. Due to the differences in questionnaire item types and scoring methods for risk perception, vaccine knowledge, and vaccine attitude, scores were calculated separately for each domain before conducting a reliability analysis. Cronbach's alpha for the combined domains was 0.49.

On COVID-19 vaccination status, participants were asked whether they were vaccinated against COVID-19 or not. Participants who had received the COVID-19 vaccine were categorized into the vaccinated group, and those who had not received the vaccine were classified as the unvaccinated group. Those who were vaccinated were further asked about the number of doses they had received, the manufacturer of the vaccines, the reasons why they wanted to be vaccinated, and whether they had any adverse reactions after vaccination. Those who had not been vaccinated were asked why they did not want to be vaccinated.

On demographic characteristics, the following information was collected: gender, age, marital status (married or not married [including unmarried, divorced, and widowed]), education level (from primary school or below to university or above), pre-retirement occupation, monthly income, place of residence (Shanghai vs. other places), region (downtown vs. countryside), and living situation (solitude vs. live with others).

To assess the perception of the risk of COVID-19, which measured individuals' perceived risk of being infected with SARS-CoV-2, considering the infectiousness of the pathogen, protection measures, and demographic status, we developed three questions: (1) “Are you concerned about the COVID-19 pandemic?” (2) “Are you confident about the prevention and control of COVID-19 epidemic?” and (3) “Do you think that older adults are more likely to be infected with SARS-CoV-2 than young ones?” We assigned a score to the answer to each of the questions, with a higher score indicating a greater perception of risk (see Appendix B).

The cognition toward COVID-19 vaccines, which measured individuals' knowledge of COVID-19, was assessed using three questions concerning vaccination procedure, age limitation for vaccination, and types of COVID-19 vaccines available in China, respectively. For each item, if participants picked the correct answer, they received a score of 1 for that question, and they received a score of 0 if they selected a wrong answer or the response of “have no idea”. The sum of the score for all three items was calculated as the total cognition score toward COVID-19 vaccines, with the total score ranging from 0 to 3. The higher score indicated that the participants had better knowledge of COVID-19 vaccines. The attitude toward COVID-19 vaccines, which measured the extent to which individuals' trust in the efficacy and safety of COVID-19 vaccines, was assessed using two questions about the efficacy and safety of COVID-19 vaccines: (1) “Do you think vaccination is an important means to prevent and control SARS-CoV-2 infection?” and (2) “Do you think COVID-19 vaccines are safe?” Each answer was scored, with a higher score indicating a more positive attitude toward the efficacy and safety of COVID-19 vaccines (see Appendix B).

The questionnaire also covered information about the duration of lockdown (from <1 week to more than 2 months), history of coronavirus infection (oneself or if relatives got infected vs. never infected), history of other vaccinations (yes vs. no), and chronic disease situation (from no chronic disease to three or more chronic diseases) of participants. How each variable was coded is displayed in Appendix B.

### Pre-testing

The questionnaire was piloted in a group comprising 10 family members of healthcare workers who were aged between 60 and 90. During the pilot, we obtained feedback from participants to identify any problematic items. After deliberation with the study investigators and participants involved in the pilot, we revised and finalized the survey items. The instrument was administered in Chinese. On average, it took participants about 15 min to complete the questionnaire.

### Recruitment process and access to questionnaire

Considering that most older adults stayed at home due to the epidemic, 90% of the questionnaires were distributed online. The link and the QR code of the online survey were sent out through a WeChat “Friends circle,” a function that can be used to share personal photos or public website links. This questionnaire link and QR code could then be forwarded or shared by participants with friends in their WeChat contact list, whom they considered appropriate for this survey; their friends were also encouraged to send the link to their friends' networks. Such a snowball sampling process continued until a sufficient sample size was reached.

It is likely that many older adults, especially those who were very old, were not good at using the Internet. To complement the data collected through online questionnaires, we also collected data through a face-to-face survey in a geriatric ward of Xinhua Hospital in Shanghai. Approximately 10% of the sample included in this study was obtained through this approach from older adults above 75 years old, with the help of a trained investigator. This, to some extent, would help mitigate the bias if all the results were from the online survey.

### Sample size computation

This is an exploratory study, and we used a cross-section design to estimate the sample size. As of 5 May 2022, 86.23% elderly population over 60 years old had been vaccinated (26). Assuming the allowable estimation error is 3%, we estimated the minimum sample size to be 507, according to the formula $=\frac{{\left(Z_{\frac{\alpha }{2}}\right)}^{2}\pi \left(1-\pi \right)}{E^{2}}$, where n is the sample size, $Z_{\frac{\alpha }{2}}$ is determined by the confidence interval, π is the overall proportion, and E is the allowable estimation error.

### Survey administration

Wen Juan Xing, an online platform similar to Amazon Mechanical Turk, Qualtrics, SurveyMonkey, or CloudResearch, was used to administer the survey results. Each participant's responses were automatically entered into the Wen Juan Xing database through a mobile phone. A dedicated link and a QR code attached to the online survey were created for the purposes of this study, and participation in the survey was voluntary. Informed consent was deemed to have been given if participants clicked on the link or scanned the QR code. There was no monetary or non-monetary incentive for completing the survey. Due to limitations of the survey platform, participants were not able to review or make changes after they submitted their responses. To prevent multiple entries from being submitted from the same individual, cookies were used to assign a unique identifier to each entry.

### Data analyses

Only completed questionnaires were analyzed in this study. Participants who terminated the survey without completing the form were deemed to have withdrawn their consent. Such data were not captured or analyzed.

A descriptive analysis was conducted for all the variables included in the analysis. The continuous variables (e.g., internal risk perception) were presented with mean and standard deviation, while categorical variables (e.g., gender) were presented with frequency. For the bivariate analysis, we compared the internal risk perception, cognition, and attitude between those who received COVID-19 vaccines and those who did not, using a *t*-test or analysis of variance (ANOVA). We also performed a path analysis to examine the association between vaccination and internal risk perception, cognition, and attitude toward COVID-19 vaccines using the AMOS software. Univariate and multivariate logistic regression models were used to examine the factors associated with COVID-19 vaccination after converting categorical variables into dummy variables. In the logistic regression models, whether the individual received COVID-19 vaccines served as the dependent variable (1 is assigned to those who were vaccinated and 0 is assigned to those who were unvaccinated). The independent variables included demographic information, internal risk perception, cognition and attitude toward COVID-19 vaccines, and individuals' health status and prior experience. Odds ratios (OR) with 95% confidence intervals (CI) were used to estimate the associations. For all the analyses, a *P* < 0.05 was considered to be statistically significant. SPSS 21.0 software was used for conducting statistical analyses.

## Results

### Demographic characteristics of the sample

The summary of participants' demographic characteristics, health status, and experience is provided in Table 1. Out of 725 participants, there were 319 male patients (44.0%) and 406 female patients (56.0%), where 465 patients (64.1%) were aged 60–70 years, and 63 patients (8.7%) were aged 80 years and above. The respondents mainly lived in Shanghai (82.1%), downtown (85.7%), had not left the community for more than 2 months (71.0%), with a high school education level or above (53.4%), were married (86.5%), lived with others (89.9%), and had an average monthly income of CNY?3,000–9,999 (77.8%). The most common occupations before retirement were workers/peasants (28.8%) and administrative staff (28.4%). A total of 199 (27.5%) of them had a history of other vaccinations. A total of 181 (25.0%) of the respondents had reported that they or their relatives had been infected with SARS-CoV-2. A total of 534 (73.7%) of the participants had chronic diseases, and 95 (13.1%) of them had three or more chronic diseases. The most common chronic diseases were hypertension, diabetes, and coronary heart disease.

**Table 1:** Sociodemographic characteristics.

**Characteristics**	**Total (*N* = 725 [%])**	**Vaccinated group (*N* = 568 [%])**	**Unvaccinated group (*N* = 157 [%])**	**χ^2^ value**
**Age (years old)**				140.65^***^
60–64	196 (27.0)	169 (29.8)	27 (17.2)	
65–69	269 (37.1)	236 (41.5)	33 (21.0)	
70–74	152 (21.0)	121 (21.3)	31 (19.7)	
75–79	45 (6.2)	27 (4.8)	18 (11.5)	
80 and above	63 (8.7)	15 (2.6)	48 (30.6)	
**Gender**				4.69^*^
Male	319 (44.0)	238 (41.9)	81 (51.6)	
Female	406 (56.0)	330 (58.1)	76 (48.4)	
**Marital status**				17.32^***^
Married	627 (86.5)	507 (89.3)	120 (76.4)	
Not married	98 (13.5)	61 (10.7)	37 (23.6)	
**Education level**				10.28^*^
Primary school and below	41 (5.7)	28 (4.9)	13 (8.3)	
Junior high school	161 (22.2)	128 (22.5)	33 (21.0)	
High school	254 (35.0)	205 (36.1)	49 (31.2)	
Junior College	133 (18.3)	111 (19.5)	22 (14.0)	
University or above	136 (18.8)	96 (16.9)	40 (25.5)	
**Pre-retirement occupation**				18.16^*^
Administrative staff	206 (28.4)	167 (29.4)	39 (24.8)	
Researcher	58 (8.0)	42 (7.4)	16 (10.2)	
Medical staff	51 (7.0)	42 (7.4)	9 (5.7)	
Police/soldier/community worker	17 (2.3)	13 (2.3)	4 (2.5)	
Educator	71 (9.8)	47 (8.3)	24 (15.3)	
Businessman/Service worker	59 (8.1)	48 (8.5)	11 (7.0)	
Worker/Peasant	209 (28.8)	161 (28.3)	48 (30.6)	
Freelancer/Unemployed	54 (7.5)	48 (8.5)	6 (3.8)	
**Monthly income (CNY)**				26.85^***^
<¥3,000	78 (10.8)	64 (11.3)	14 (8.9)	
¥3,000–5,999	343 (47.3)	274 (48.2)	69 (43.9)	
¥6,000–9,999	221 (30.5)	183 (32.2)	38 (24.2)	
¥10,000–14,999	61 (8.4)	35 (6.2)	26 (16.6)	
¥15,000 and above	22 (3.0)	12 (2.1)	10 (6.4)	
**Place of residence**				9.56^**^
Shanghai	595 (82.1)	453 (79.8)	142 (90.4)	
Other cities	130 (17.9)	115 (20.2)	15 (9.6)	
**Region**				0.02
Downtown	621 (85.7)	487 (85.7)	134 (85.4)	
Countryside	104 (14.3)	81 (14.3)	23 (14.6)	
**Living situation**				6.03^*^
Solitude	73 (10.1)	49 (8.6)	24 (15.3)	
Live with others	652 (89.9)	519 (91.4)	133 (84.7)	
**Time of lockdown**				2.72
<1 week	69 (9.5)	55 (9.7)	14 (8.9)	
1 week−1 month	46 (6.3)	40 (7.0)	6 (3.8)	
1 month−2 months	95 (13.1)	76 (13.4)	19 (12.1)	
>2 months	515 (71.0)	397 (69.9)	118 (75.2)	
**Coronavirus infection**				2.28
Infected (oneself/relatives)	181 (25.0)	149 (26.2)	32 (20.4)	
Never infected	544 (75.0)	419 (73.8)	125 (79.6)	
**Other vaccines**				14.88^***^
Yes	199 (27.5)	175 (30.8)	24 (15.3)	
No	526 (72.6)	393 (69.2)	133 (84.7)	
**Chronic disease**				31.37^***^
1 chronic disease	315 (43.5)	258 (45.4)	57 (36.3)	
2 chronic diseases	124 (17.1)	86 (15.1)	38 (24.2)	
3 or more chronic diseases	95 (13.1)	47 (8.3)	48 (30.6)	
No chronic disease	191 (26.3)	177 (31.2)	14 (8.9)	

^*^P < 0.05, ^**^P < 0.01, and ^***^P < 0.001.

### Vaccination status

**Figure 1:**
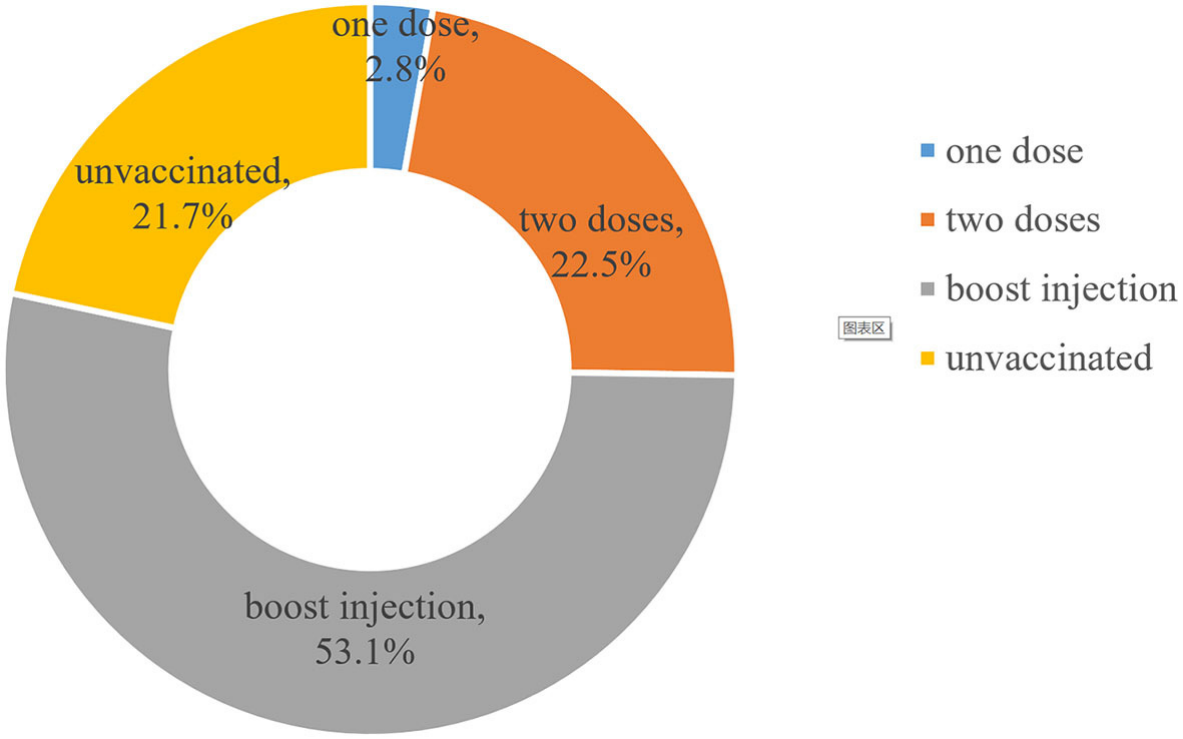
Vaccination status among older adults (N = 725).

**Figure 2:**
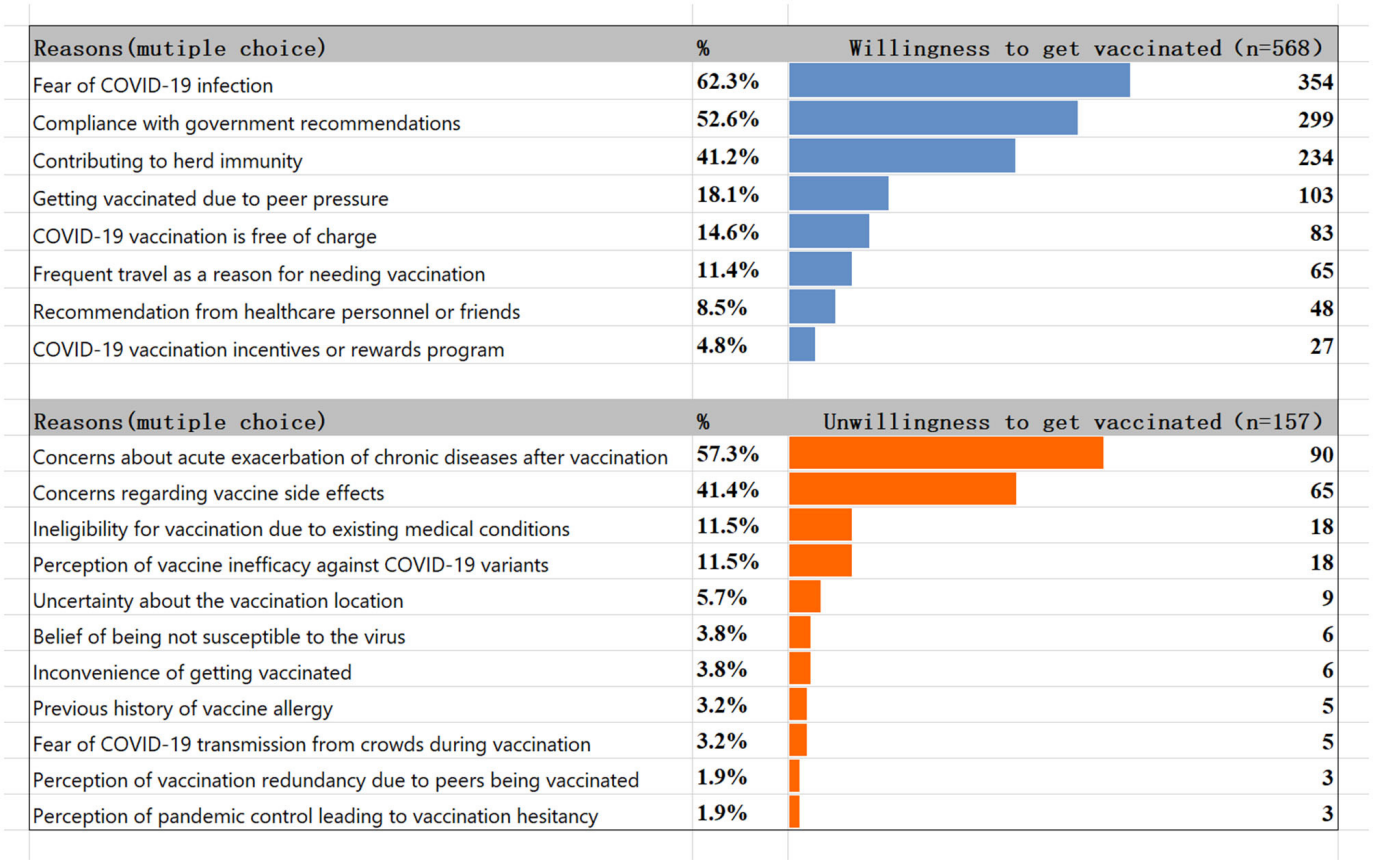
Reasons for willingness and unwillingness to get vaccinated.

Out of 725 individuals, 568 were vaccinated against COVID-19, with a vaccination rate of 78.3%. A total of 385 (53.1%) received booster injections, 163 (22.5%) received two doses, and 20 (2.8%) received only one dose (see Figure 1). The main vaccines were the Sinovac COVID-19 vaccine (65.5%) and the Sinopharm COVID-19 vaccine (BIBP) (23.2%). The top three self-reported reasons to get vaccinated (multiple selections) were “fear of COVID-19 infection (62.3%)”, “compliance with government recommendations (52.6%)”, and “contributing to herd immunity (41.2%)”. Self-reported reasons for unwillingness to get vaccinated (multiple selections) were “concerns about acute exacerbation of chronic diseases after vaccination (57.3%)”, “concerns about the vaccine's side effects (41.4%)”, “ineligibility for vaccination due to existing medical conditions (11.5%)”, and “perception of vaccine inefficacy against COVID-19 variants (11.5%)” (see Figure 2).

### Internal risk perception, cognition, and attitude toward COVID-19 vaccines

The average score of internal risk perception was 2.22 ± 0.31 in the vaccinated group and 2.14 ± 0.36 in the unvaccinated group. There was a statistically significant difference between the two groups (*t* = 2.64, *P* < 0.05). The cognition score of COVID-19 vaccines in the vaccinated group was 1.55 ± 0.67 compared to 1.13 ± 0.83 in the unvaccinated group. The difference was statistically significant between the two groups (*t* = 5.84, *P* < 0.05). The average score of attitudes toward COVID-19 vaccines was 1.81 ± 0.29 in the vaccinated group as compared to 1.56 ± 0.38 in the unvaccinated group. The difference was statistically significant between the two groups (*t* = 7.92, *P* < 0.05) (see Table 2).

**Table 2:** Comparison of internal risk perception, cognition, and attitude toward COVID-19 vaccine.

**Item**	**Scores of vaccinated group**	**Sores of unvaccinated group**	***t*-value**
**Internal risk perception**	2.22 ± 0.31	2.14 ± 0.36	2.64^**^
1. Attention to the epidemic	0.82 ± 0.18	0.74 ± 0.22	4.58^***^
2. Confidence in epidemic prevention control	0.55 ± 0.15	0.57 ± 0.17	−1.42
3. Older adults are more likely to be infected than the young ones	0.85 ± 0.20	0.83 ± 0.23	1.30
**Cognition toward COVID-19 vaccine**	1.55 ± 0.67	1.13 ± 0.83	5.84^***^
1. Vaccination procedure	0.88 ± 0.33	0.63 ± 0.48	5.95^***^
2. Age limitation for vaccination	0.62 ± 0.49	0.48 ± 0.50	3.24^**^
3. Types of COVID-19 vaccines	0.05 ± 0.22	0.02 ± 0.14	2.12^*^
**Attitude toward COVID-19 vaccine**	1.81 ± 0.29	1.56 ± 0.38	7.92^***^
1. Efficacy of COVID-19 vaccine	0.93 ± 0.17	0.81 ± 0.24	5.90^***^
2. Safety of COVID-19 vaccine	0.88 ± 0.17	0.78 ± 0.19	8.51^***^

^*^P < 0.05, ^**^P < 0.01, and ^***^P < 0.001.

**Figure 3:**
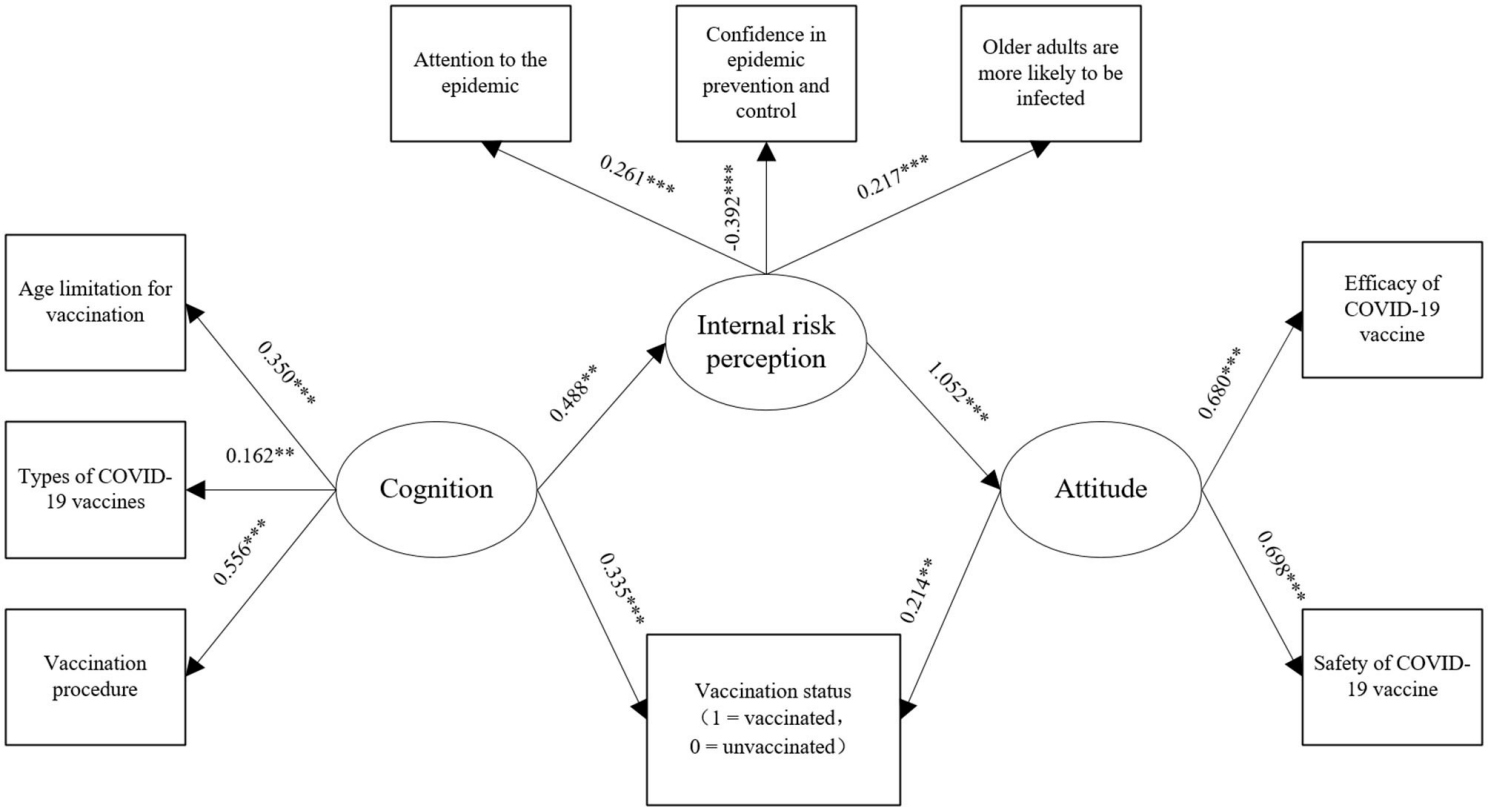
Path analysis of vaccination with internal risk perception, cognition, and attitude. **P < 0.01 and ***P < 0.001.

Path analysis showed that the model fitted well (*X*^2^/df = 3.55, NFI = 0.86, IFI = 0.90, CFI = 0.89, and RMSEA = 0.06). From the total effects of various influencing factors on the vaccination behavior of older adults, we found that (1) the cognitive effect on vaccination behavior was relatively large, followed by the internal risk perception, and then the attitude toward COVID-19 vaccines; (2) cognition had a positive effect on vaccination behavior (with a total effect of 0.44, direct effect of 0.34, and indirect effect of 0.11). The higher the cognition of COVID-19 vaccines was, the more likely older adults are to receive the COVID-19 vaccines. Moreover, cognition successively affects vaccination behavior through internal risk perception with a direct effect of 0.49 and attitude with an indirect effect of 0.51; and (3) intrinsic risk perception had a positive effect on vaccination behavior with an indirect effect of 0.23. The higher the intrinsic risk perception of COVID-19, the more likely older adults received the vaccination, and this effect was generated through attitude with a direct effect of 1.05. (4) The attitude toward COVID-19 vaccines had a positive effect on vaccination behavior with a direct effect of 0.21. The more positive the attitude toward COVID-19 vaccines was, the more likely older adults were vaccinated (see Figure 3).

### Univariate and multivariate logistic analyses of vaccination status in older adults

The bivariate logistic analysis shows that (Table 3, unadjusted odds ratio) age (OR = 0.48, *P* < 0.001), gender (OR = 0.68, *P* < 0.05), marital status (OR = 2.56, *P* < 0.001), monthly income (OR = 0.72, *P* < 0.01), place of residence (OR = 0.42, *P* < 0.01), living situation (OR = 0.52, *P* < 0.05), history of other vaccines (OR = 2.47, *P* < 0.001), number of chronic diseases (OR = 0.44, *P* < 0.001), internal risk perception (OR = 2.18, *P* < 0.01), cognition toward COVID-19 (OR = 2.18, *P* < 0.001), and attitude toward COVID-19 (OR = 9.01, *P* < 0.001) were associated with vaccination status in older adults (for more details, please see Appendix C).

**Table 3:** Multivariate logistic regression analysis of COVID-19 vaccination in older adults.

**Variables**	**Unadjusted odds ratio (OR)**	**Adjusted odds ratio (OR)**	**95%CI for adjusted OR**
Age	0.48^***^	0.53^***^	0.43–0.66
Gender (male = 1)	0.68^*^	1.08	0.64–1.80
Marital status (married = 1)	2.56^***^	1.64	0.85–3.16
**Education levels**			
**Primary school and below (Reference)**			
Junior high school	0.90	0.77	0.19–3.04
High school	1.62	1.09	0.42–2.79
Junior College	1.74^*^	0.70	0.32–1.53
University or above	2.10^*^	1.00	0.44–2.30
**Pre-retirement occupation**			
**Administrative staff (reference)**			
Researcher	0.54	0.52	0.17–1.60
Medical staff	0.33^*^	0.52	0.14–2.02
Police/soldier/community worker	0.58	0.61	0.14–2.66
Educator	0.41	0.22	0.04–1.24
Businessman/service worker	0.25^**^	0.52	0.13–2.01
Worker/peasant	0.55	0.38	0.10–1.37
Freelancer/unemployed	0.42	0.36	0.12–1.11
Monthly income	0.72^**^	0.91	0.66–1.28
Place of residence (Shanghai = 1)	0.42^**^	0.40	0.17–0.92
Region (downtown = 1)	1.03	1.22	0.60–2.50
Living situation (solitude = 1)	0.52^*^	0.73	0.35–1.54
**Time of lockdown**			
<**1 week (Reference)**			
1 week−1 month	1.17	0.33^*^	0.13–0.83
1 month−2 months	1.98	0.67	0.21–2.12
>2 months	1.19	0.62	0.31–1.25
Coronavirus infection (Infected = 1)	1.39	1.30	0.75–2.24
History of other vaccines (Prior vaccination = 1)	2.47^***^	2.58^***^	1.45–4.60
Number of chronic diseases	0.44^***^	0.49^***^	0.38–0.62
Internal risk perception	2.18^**^	1.48	0.75–2.90
Cognition	2.18^***^	1.60^**^	1.17–2.19
Attitude	9.01^***^	9.22^***^	4.69–18.09

^*^P < 0.05, ^**^P < 0.01, and ^***^P < 0.001.

The multivariable logistic analysis shows that age, measured as the continuous variable, was identified as a significant impact factor, and older respondents were less likely to accept COVID-19 vaccines (OR = 0.53, 95% CI 0.43–0.66, *P* < 0.001). A high level of cognition and a more positive attitude toward COVID-19 vaccines were important factors associated with the COVID-19 vaccination. Older adults with a high level of cognition toward COVID-19 vaccines were more willing to receive the vaccine than those who had a lower level of cognition (OR = 1.60, 95% CI 1.17–2.19, *P* < 0.01). Older adults with a more positive attitude toward COVID-19 vaccines were more willing to receive the vaccine than those with a positive attitude (OR = 9.22, 95% CI 4.69–18.09, *P* < 0.001). The respondents who lived in Shanghai were less likely to be vaccinated than those who lived in other cities (OR = 0.40, 95% CI 0.17–0.92, *P* < 0.05). Older adults with a lockdown period between 1 week and 1 month were less likely to be vaccinated than those who had a shorter lockdown period of <1 week (OR = 0.33, 95% CI 0.13–0.83, *P* < 0.05). Older adults who had a history of other vaccines were more likely to be vaccinated than those who had never obtained other vaccines (OR = 2.58, 95% CI 1.45–4.60, *P* < 0.01). Older adults with more numbers of chronic diseases were less likely to be vaccinated than those with fewer numbers of chronic diseases (OR = 0.49, 95% CI 0.38–0.62, *P* < 0.001) (see Table 3).

## Discussion

To the best of our knowledge, this is the first study that evaluates COVID-19 vaccination coverage and its cognitive determinants among older adults in China. The study shows that 78.3% (568/725) of older adults received COVID-19 vaccines, and slightly more than half of them had booster shots. Concerns about chronic disease and adverse reactions were major reasons for being unwilling to be vaccinated among older adults. Our study adds to the prevailing global evidence that the factors such as age, time of lockdown, a history of other vaccines, and number of chronic diseases were associated with COVID-19 vaccine hesitancy among older adults, which mirrored the findings from other studies (27–29). Older adults with a high level of cognition and a more positive attitude toward COVID-19 vaccines were more willing to receive the vaccine.

Older adults have a higher infection risk and suffer more severe health consequences if infected. Therefore, they are the priority group for vaccination in many countries and territories (30, 31). Studies conducted in other countries found higher acceptability of COVID-19 vaccines among the older population (32, 33), and the full vaccination rate among people aged 60 years and older has reached 85–90% in many Western countries (34). However, some studies in China indicate that older adults are less willing to accept COVID-19 vaccination compared to those aged 18–59 years (35, 36). This could be due to the low morbidity of older adults at the beginning of the epidemic when they were less exposed to the infection risk during a well-controlled phase of the pandemic, making it harder to raise their awareness about the importance of vaccination. In China, people above 60 years of age were excluded from the first batch of eligible populations to receive vaccination due to uncertainty on health risks that may be resulted from the newly developed vaccines. This factor has resulted in low COVID-19 vaccine coverage among older adults in China. Despite the availability of COVID-19 vaccines for mass vaccination as early as December 2020 (37), older adults were not recommended to receive vaccination until March 2021 (38).

Our study found a clear downward trend in the vaccination rate for older adults with increasing age. People under the age of 70 years generally have better health, more contact with others, and are more active in receiving COVID-19 vaccines. However, as age increases, older adults tend to have more chronic diseases, restricted mobility, and reduced contact with the outside world. In some cases, older adults and their families may even believe that vaccination is unnecessary if they do not leave their homes frequently. This poses significant challenges in increasing vaccination coverage among old adults to reach a high level of herd immunity. To address this issue, more resources and specifically designed interventions are needed to motivate older adults and their families to get vaccinated.

Our study found that a good understanding and sound knowledge of COVID-19 vaccines were positively associated with vaccination. Cognition is a key determinant of individuals' behavior. It can prompt individuals to act and encourage them to take suitable measures (39). The higher level of the cognition of COVID-19 vaccines, the more likely older adults are to receive COVID-19 vaccines. Moreover, cognition is found to successively affect vaccination behavior through internal risk perception and attitude. Thus, providing accurate information to older adults is critical. Compared to young adults, older adults tend to have fewer sources of information on COVID-19 vaccination and rely more on traditional media, family, friends, and health workers for vaccination recommendations. Social media has played an indispensable role in informing the public about COVID-19 vaccination, particularly during the lockdown period (40). Therefore, authorities should release the latest information on both new and traditional social media platforms to highlight the potential benefits of obtaining COVID-19 vaccines for older adults. Additionally, health workers' recommendations are a vital source of information that can influence patients' vaccination willingness (41). Therefore, health workers should actively guide older adults to get vaccinated.

Similar to many studies (42, 43), we also found a positive relationship between the perception of the safety and efficacy of a vaccine and individuals' acceptance of the vaccine, especially for a newly developed vaccine such as the COVID-19 vaccine. Older adults with more positive attitudes toward COVID-19 vaccines were more likely to be vaccinated. The reason for the negative attitude toward the COVID-19 vaccine was the doubt of safety and efficacy. Compared to many established vaccines, public confidence in the safety and efficacy of COVID-19 vaccines was low (44). Many older adults believed that the vaccine's safety and efficacy could not be guaranteed. The doubt arises from (1) the development of a vaccine for a new pathogen having been pushed much faster than ever before and (2) new bioscience technologies (e.g., mRNA vaccine) being used in humans for the first time (45). Although the safety and efficacy of COVID-19 vaccination have been gradually verified with the increasing number of people vaccinated against COVID-19 worldwide, the relatively high frequency of side effects of the vaccination continues casting doubts on the safety and efficacy of the COVID-19 vaccines. China reported the incidence of adverse reactions of 11.86/100,000 doses during the period of 15 December 2020–30 April 2021 (46). Making vaccine clinical trial data open and transparent is an effective way to address public skepticism. This includes explaining how vaccines work and how they are developed, from recruitment to regulatory approval based on safety and efficacy, to enhance the trust in vaccines in older adults. Additionally, effective campaigns should also be carried out to provide information on the effectiveness of vaccines, the duration of the vaccine protection, and the importance of population-wide coverage to achieve herd immunity.

Older adults often suffer from multiple chronic diseases. The number of chronic diseases is correlated with vaccination status. We found that older adults classified as having poor health displayed a higher level of COVID-19 vaccine hesitancy. The lower coverage among them is likely due to two major reasons. First, older adults with poor health are more concerned about the side effect of the vaccines, which is understandable since some vaccines were reported to be unsuitable for select populations due to relatively poor immunity function or allergic predisposition. Second, they may have faced delays in receiving COVID-19 vaccines. When the COVID-19 vaccine was first introduced in China, it was not recommended to be used for people with certain conditions (47). However, a study showed that adults aged 60 years and above did not have a significantly higher risk of adverse events after the Coronavirus vaccination compared to the baseline period (48). The COVID-19 vaccination still shows significant benefits in reducing COVID-19 incidence and deaths in places where COVID-19 was prevalent. Such information should be reached to older adults with chronic illnesses.

### Limitations

Several limitations need to be acknowledged. First, our study has a small sample size, and the data were primarily collected through an online survey platform. Therefore, the sample may not represent the geographic, cultural, and socioeconomic variations among Chinese older adults. Second, this is a cross-sectional study, which means that we cannot determine the causality of COVID-19 vaccinations and associated factors. Third, the online questionnaire is self-reported and may have a certain degree of information deviation. However, the online anonymous questionnaire is more helpful in obtaining information on sensitive topics than the face-to-face survey. Fourth, the reliability of the questionnaire remains low. One of the possible reasons for this low reliability is that we introduced different types of requestions and different numbers of options for questions in the questionnaire in the hope that this would allow the questionnaire to be easier for old adults to understand and facilitate the completion of the questionnaire. In future questionnaire design, we will seek to strike a balance between the cognitive abilities of older adults and the appropriate question types and number of options. Despite these limitations, this study provides important information on factors associated with COVID vaccination among older adults, whose protection is key to reducing COVID-19-related deaths.

## Conclusion

The battle against the COVID-19 pandemic is not over, and implementing appropriate prevention and control measures in older adults remains critical. The COVID-19 vaccine has been shown to reduce the risk of SARS-CoV-2 transmission, decrease the risk of hospitalization, and promote safety in older adults. Factors such as age, duration of lockdown, history of other vaccines, numbers of chronic diseases, cognition, and attitude toward COVID-19 vaccines are important factors associated with the COVID-19 vaccination. Disseminating accurate information on COVID-19 vaccines to improve awareness, cognition, and attitude toward COVID-19 vaccines would help improve vaccination coverage among older adults. Government authorities can use social media platforms to provide scientific information on the safety and efficacy of COVID-19 vaccines.

## Data availability statement

The original contributions presented in the study are included in the article/Supplementary material, further inquiries can be directed to the corresponding authors.

## Ethics statement

The studies involving human participants were reviewed and approved by Ethics Committee of Xinhua Hospital Affiliated to Shanghai Jiao Tong University School of Medicine. The patients/participants provided their written informed consent to participate in this study.

## Author contributions

LW and WZ performed data collection and analysis and drafted the manuscript. YH, GY, and YC were all responsible for participant recruitment and data collection. LY and YC designed and edited the study. All authors read and approved the final manuscript.
