# Supplementary material for: COVID-19 vaccination coverage and its cognitive determinants among older adults in Shanghai, China, during the COVID-19 epidemic
Source: Front Public Health. 2023 Jun 2;11:1163616. doi: 10.3389/fpubh.2023.1163616 (PMC10272832; doi:10.3389/fpubh.2023.1163616)
Supplement: Supplementary file 1 [file Data_Sheet_1.pdf]

Supplementary materials

Appendix A

Investigation on COVID-19 vaccination among elder adults

1. Age (years old)

□ 60 to 64 □ 65 to 69 □ 70 to 74 □ 75 to 79 □ 80 and above

2. Gender

□ Male □ female

3. Marital status

□ Married □ Not married (including unmarried, divorced, and widowed)

4. Education level

□ Primary School and below □ Junior high school □ High School

□ Junior College □ University or above

5. Pre-retirement occupation

□ Administrative staff □ Researcher □ Medical staff

□ Police/Soldier/Community worker □ Educator □ Businessman/Service workers

□ Worker/Peasant □ Freelancer/Unemployed

6. Are you still working?

□ Retired, not working □ Not retired, working □ Working after retirement

7. What is your monthly pension or disposable income? (CNY)

□ Less than 3000 Yuan □ 3000 to 5999 yuan □ 6000 to 9999 Yuan

□ 10000 to 14,999 Yuan □ 15000 yuan and above

8. Which city do you live in?

□ Shanghai □ Not Shanghai

9. Where do you live?

□ Downtown □ Countryside

10. What is your living situation?

□ Solitude □ Live with others

11. What is your physical condition?

□ Very Healthy □ Good □ Average □ Poor

12. Do you suffer from any of the following diseases? (multiple choice)

□ Hypertension □ diabetes □ Coronary heart disease □ chronic bronchitis

□ Cerebrovascular disease □ Autoimmune diseases □ Hematological disease

□ Malignant tumors □ Other chronic diseases □ None of these diseases

13. Are you concerned about the COVID-19 pandemic?

□ Very concerned □ Relatively concerned □ Generally concerned □ Not concerned

14. Are you confident about the prevention and control of COVID-19 epidemic?

□ Optimistic □ Pessimistic

15. How has COVID-19 affected your life?

□ Very large effect □ Relatively large effect □ Moderate effect

□ Relatively small effect □ No effect

16. How long did you lockdown in your community or residence during the epidemic?

□ Less than 1 week □ Less than 1 month □1 month to 2 months

□ More than 2 months

17. What do you often do during the epidemic? (multiple choice)

□ Reading, watching TV or watching mobile phones □ Online shopping
□ Chatting and making phone calls with friends and relatives
□ Doing exercises, doing housework, cooking, growing flowers or knitting
□ Taking care of grandchildren □ Taking part in epidemic prevention work (eg. Community volunteers) □ Still working □ Sleeping □ Doing nothing

18. Do you think older adults are more likely to be infected SARS-CoV-2 than the young ones?
□ More likely □ Less likely □ Equally likely

19. Have you or your family been infected with SARS-CoV-2COVID-19?
□ Infected □ Never infected

20. Do you think vaccination is an important means to prevent and control SARS-CoV-2 infection?
□ Yes □ No

21. Do you think COVID-19 vaccines are safe?
□ Safe □ Unsafe □ Hard to say

22. Do you know what the age limit is for COVID-19 vaccination?
□ Less than 60 years old □ Less than 70 years old □ Less than 80 years old
□ No upper age limit □ Have no idea

23. Do you know what kinds of COVID-19 vaccines are available in China? (multiple choice)
□ Inactivated vaccine □ Adenovirus vector vaccine □ Genetically engineered recombinant protein vaccine □ Nucleic acid vaccine □ Live attenuated vaccine
□ Not clear

24. Do you know the procedure of inactivated COVID-19 vaccine in China?
□ 1 dose per year □ Just one dose □ 2 doses (3-8 weeks apart) + 1 booster shot (after 6 months) □ Have no idea

25. Have you received any of the following unscheduled vaccines? (multiple choice)
□ Influenza vaccine □ Pneumonia vaccine □ Recombinant herpes zoster vaccine
□ None

26. Have you been vaccinated against COVID-19?
□ 1 dose □ 2 doses □ 2 doses +1 booster (3 doses in total) □ Not vaccinated

If you have been vaccinated, do the item 27, 28, 29.
If you have not been vaccinated, do the item 30.

27. Which of the following vaccines have you received? (multiple choice)
□ Beijing Sinovac inactivated coronavirus vaccine □ Sinopharm Beijing Biological Coronavirus inactivated vaccine □ Sinopharm Wuhan biological coronavirus inactivated vaccine
□ Anhui Zhifei Biological Recombinant Subunit vaccine
□ Conhino virus vector vaccine □ Other ________ □ Not clear

28. Why do you want to get vaccinated against COVID-19? (multiple choice)
□ Fear of COVID-19 infection □ Recommendation from healthcare personnel or friends
□ Frequent travel as a reason for needing vaccination □ COVID-19 vaccination is free of charge
□ Compliance with government recommendations □ Contributing to herd immunity
□ Getting vaccinated due to peer pressure □ COVID-19 vaccination incentives or rewards program

29. Have you suffered any of the following symptoms within one week of receiving COVID-19 vaccine? (multiple choice)
□ Pain at injection site, rash □ Cough, runny nose, sore throat
□ Fatigue, muscle soreness □ Nausea, vomiting, abdominal pain, diarrhea

□ Dizziness, headache □ Fever □ Other _____ □ No discomfort

30. Why have you not been vaccinated against COVID-19? (multiple choice)

□ Concerns about acute exacerbation of chronic diseases after vaccination

□ Concerns regarding vaccine side effects □ Belief of being not susceptible to the virus

□ Ineligibility for vaccination due to existing medical conditions □ Previous history of vaccine allergy

□ Perception of vaccine inefficacy against COVID-19 variants

□ Perception of vaccination redundancy due to peers being vaccinated

□ Perception of pandemic control leading to vaccination hesitancy

□ Inconvenience of getting vaccinated □ Uncertainty about the vaccination location

□ Fear of COVID-19 transmission from crowds during vaccination

Appendix B

TABLE B1 Questions and scores of each answer.

Question	Answer	Scores of each answer
Q1. Are you concerned about the COVID-19 pandemic?	A1. Very concerned	1.00
	A2. Relatively concerned	0.75
	A3. Generally concerned	0.50
	A4. Not concerned	0.25
Q2. Are you confident about the prevention and control of COVID-19 epidemic?	A1. Optimistic	0.50
	A2. Pessimistic	1.00
Q3. Do you think older adults are more likely to be infected SARS-CoV-2 than the young ones?	A1. More likely	1.00
	A2. Less likely	0.33
	A3. Equally likely	0.66
Q4. Do you know the procedure of inactivated COVID-19 vaccine in China?	A1. 1 dose per year	0.00
	A2. Just one dose	0.00
	A3. 2 doses (3-8 weeks apart) and 1 booster shot (after 6 months)	1.00
	A4. Have no idea	0.00
Q5. Do you know what the age limit is for COVID-19 vaccination?	A1. Less than 60 years old	0.00
	A2. Less than 70 years old	0.00
	A3. Less than 80 years old	0.00
	A4. There is no upper age limit	1.00
	A5. Have no idea	0.00
Q6. Do you know the types of domestic COVID-19 vaccines?(multiple choice)	A1. Correct answer	1.00
	A2. Incorrect answer	0.00
Q7. Do you think vaccination is an important means to prevent and control SARS-CoV-2 infection?	A1. Yes	1.00
	A2. No	0.50
Q8. Do you think COVID-19 vaccines are safe?	A1. Safe	1.00
	A2. Unsafe	0.33
	A3. It’s hard to say	0.66

TABLE B2 Variable assignment.

Item	Variable	Code
Age（years old）	60-64	1
	65-69	2
	70-74	3
	75-79	4
	80 and above	5
Gender	Male	1
	Female	0
Marital status	Married	1
	Not married	0
Education level	Primary school and below	1
	Junior high school	2
	High school	3
	Junior College	4
	University or above	5
Pre-retirement occupation	Administrative staff	1
	Researcher	2
	Medical staff	3
	Police/Soldier/Community worker	4
	Educator	5
	Businessman/Service worker	6
	Worker/Peasant	7
	Freelancer/Unemployed	8
Monthly income (CNY)	Less than ￥3000	1
	￥3000-5999	2
	￥6000-9999	3
	￥10000-14999	4
	￥15000 and above	5
Place of residence	Shanghai	1
	Other cities	0
Region	Downtown	1
	Countryside	0
Living situation	Solitude	1
	Live with others	0
Time of lockdown	Less than 1 week	1
	1 week to 1 month	2
	1 month to 2 months	3
	More than 2 months	4
History of SARS-CoV-2 infection	Infected(oneself/relatives)	1
	Never infected	0
History of other vaccines	Yes	1
	No	0

Chronic disease	1 chronic disease	1
	2 chronic diseases	2
	3 or more chronic diseases	3
	No chronic disease	0

Appendix C

TABLE C1 Univariate logistic regression analysis of COVID-19 vaccination in older adults.

**Variables**	**OR**	**95%CI**	***P*-value**
**Age**	0.48	0.41-0.56	<0.001
**Gender(Male=1)**	0.68	0.48-0.97	0.031
**Marital status(Married=1)**	2.56	1.63-4.04	<0.001
**Education levels**			0.039
Primary school and below (Reference)			
Junior high school	0.90	0.42-1.91	0.779
High school	1.62	0.95-2.75	0.077
Junior College	1.74	1.08-2.83	0.024
University or above	2.10	1.17-3.78	0.013
**Pre-retirement occupation**			0.089
Administrative staff (reference)			
Researcher	0.54	0.21-1.34	0.182
Medical staff	0.33	0.12-0.92	0.033
Police/Soldier/Community worker	0.58	0.19-1.78	0.343
Educator	0.41	0.04-1.24	0.209
Businessman/Service worker	0.25	0.10-1.66	0.005
Worker/Peasant	0.55	0.09-0.65	0.268
Freelancer/Unemployed	0.42	0.19-1.59	0.061
**Monthly income**	0.72	0.59-0.87	0.001
**Place of residence (shanghai = 1)**	0.42	0.24-0.74	0.003
**Region (downtown = 1)**	1.03	0.63-1.70	0.902
**Living situation (Solitude = 1)**	0.52	0.31-0.88	0.015
**Time of lockdown**			0.449
Less than 1 week (Reference)			
1 week to 1 month	1.17	0.63-2.17	0.625
1 month to 2 months	1.98	0.82-4.79	0.129
More than 2 months	1.19	0.69-2.05	0.532
**Coronavirus infection (Infected = 1)**	1.39	0.90-2.14	0.135
**History of other vaccines (Prior vaccination = 1)**	2.47	1.54-3.95	<0.001
**Number of chronic diseases**	0.44	0.37-0.54	<0.001
**Internal risk perception**	2.18	1.27-3.76	0.005
**Cognition**	2.18	1.71-2.80	<0.001
**Attitude**	9.01	5.33-15.23	<0.001
